# Exposure to phenytoin associates with a lower risk of post-COVID cognitive deficits: a cohort study

**DOI:** 10.1093/braincomms/fcac206

**Published:** 2022-08-16

**Authors:** Maxime Taquet, Paul J Harrison

**Affiliations:** Department of Psychiatry, University of Oxford, Oxford, U.K; Oxford Health NHS Foundation Trust, Oxford, U.K; Department of Psychiatry, University of Oxford, Oxford, U.K; Oxford Health NHS Foundation Trust, Oxford, U.K

**Keywords:** RIPK signaling, Endotheliopathy, Mpro, brain fog, Long COVID

## Abstract

Post-COVID cognitive deficits (often referred to as ‘brain fog’) are common and have large impacts on patients’ level of functioning. No specific intervention exists to mitigate this burden.

This study tested the hypothesis, inspired by recent experimental research, that post-COVID cognitive deficits can be prevented by inhibiting receptor-interacting protein kinase (RIPK). Using electronic health record data, we compared the cognitive outcomes of propensity score-matched cohorts of patients with epilepsy taking phenytoin (a commonly used RIPK inhibitor) vs. valproate or levetiracetam at the time of COVID-19 diagnosis.

Patients taking phenytoin at the time of COVID-19 were at a significantly lower risk of cognitive deficits in the six months after COVID-19 infection than a matched cohort of patients receiving levetiracetam (hazard ratio 0.78, 95% CI 0.63-0.97, p = 0.024) or valproate (hazard ratio 0.73, 95% CI 0.58-0.93, p = 0.011). In secondary analyses, results were robust when controlling for subtype of epilepsy, and showed specificity to cognitive deficits in that similar associations were not seen with other ‘long-COVID’ outcomes such as persistent breathlessness or pain.

These findings provide pharmacoepidemiological support for the hypothesis that RIPK signaling is involved in post-COVID cognitive deficits. These results should prompt empirical investigations of RIPK inhibitors in the prevention of post-COVID cognitive deficits.

**Running title**: Phenytoin and post-COVID cognitive deficit

**Abbreviations:** BMI = Body mass index; EHR = Electronic Health Records; HR = Hazard ratio; MCI = Mild cognitive impairment; NF = Nuclear factor; NEMO = NF-κB essential modulator; RIPK = receptor-interacting protein kinase; SMD = standardised mean difference

## Introduction

A large proportion of patients experience long-lasting symptoms in the weeks and months after a diagnosis of COVID-19.[1–3] Of those symptoms, cognitive impairment (also referred to as "brain fog") is particularly worrisome: it is one of the most common,[4,5] can affect those with even relatively mild acute COVID-19 illness,[1,5] and results in the inability to work for many affected patients.[3] While emerging research is starting to characterise the clinical presentation of post-COVID cognitive deficits,[6] its pathogenesis remains elusive. Identifying therapeutic targets is critical to reducing the burden of this COVID-19 complication.

Endotheliopathy has been hypothesised as one potential mechanism underlying post-COVID cognitive deficits.[7] According to recent research, microvascular brain pathology following COVID-19 can be caused by SARS-CoV-2 main protease $M^{pro}$ cleaving nuclear factor (NF)-κB essential modulator (NEMO) thus inducing the death of brain endothelial cells.[8] The same study showed that pharmacologically inhibiting receptor-interacting protein kinase (RIPK) signaling prevents the $M^{pro}$-induced microvascular pathology.[8]

This research leads to the following hypothesis: exposure to a pharmacological inhibitor of RIPK signaling at the time of COVID-19 infection reduces the risk of post-COVID cognitive deficits. In this study, we tested this hypothesis using a retrospective cohort study based on electronic health records data. While many pharmacological agents inhibit RIPK signaling,[9] most are only used in very rare clinical scenarios (e.g. sunitinib for the treatment of advanced renal cell carcinoma or pancreatic neuroendocrine tumours). The exception is phenytoin which is used as an anti-epileptic drug and which, amongst its other effects, is a RIPK1 inhibitor protecting against necroptosis.[10,11]

In this study, we compared the incidence of post-COVID cognitive deficits between patients exposed to phenytoin and matched cohorts of patients exposed to other anti-epileptic drugs at the time of their COVID-19 diagnosis.

## Materials and Methods

### Data

The study used TriNetX Analytics, a federated network of linked EHRs recording anonymized data from 59 healthcare organizations (HCOs), primarily in the USA, totalling 81 million patients. Available data include demographics, diagnoses (using ICD-10 codes), procedures (using CPT codes), medications (encoded as RxNorm codes and VA classes), and measurements (e.g. BMI). The HCOs in the network are a mixture of hospitals, primary care, and specialist providers, and they contribute data from uninsured and insured patients. Data de-identification is formally attested as per Section §164.514(b)(1) of the HIPAA Privacy Rule, superseding TriNetX's waiver from the Western Institutional Review Board; no further ethical approval was thus needed. As the study uses fully anonymised routinely collected data, no consent from participants was required. Within TriNetX, cohorts can be defined based on inclusion and exclusion criteria, propensity score-matched on a set of covariates, and the outcomes can be compared between matched cohorts. For further details about TriNetX, its data provenance and functionalities, see Supplement.

### Cohorts

The primary cohort was defined as all individuals who met the following 3 criteria:

A. The individual has a diagnosis of epilepsy or recurrent seizures (ICD-10 code G40)

B. The individual has a confirmed diagnosis of COVID-19 or a positive PCR test for SARS-CoV-2 between January 20, 2020 (date of the first recorded COVID-19 case in the USA) and November 29, 2021.

C. The individual has phenytoin recorded in their medications on the day of their COVID-19 diagnosis or positive PCR test for SARS-CoV-2 or within 3 months before it.

Two control cohorts were defined. They both also met inclusion criteria A and B, but instead of C, they had to meet C' (first control cohort) or C" (second control cohort):

C'. The individual has levetiracetam recorded in their medications on the day of their COVID-19 diagnosis or positive PCR test for SARS-CoV-2 or within 3 months before it.

C''. The individual has valproate recorded in their medications on the day of their COVID-19 diagnosis or positive PCR test for SARS-CoV-2 or within 3 months before it.

In addition, individuals who had any record of receiving phenytoin during the study period or up to 6 months before the start of the study period (January 20, 2020) were excluded from both control cohorts.

Details of the cohort definition including EHR codes can be found in the Supplement.

### Covariates

As in our previous studies, a set of risk factors for COVID-19 and for more severe COVID-19 illness was used[1]: age, sex, race, ethnicity, obesity, hypertension, diabetes, chronic kidney disease, asthma, chronic lower respiratory diseases, nicotine dependence, substance misuse, previous psychiatric illness, ischaemic heart disease and other forms of heart disease, socioeconomic deprivation, cancer (and haematological cancer in particular), chronic liver disease, stroke, dementia, organ transplant, rheumatoid arthritis, lupus, psoriasis, and disorders involving an immune mechanism. In addition, because of the comorbidity between epilepsy and mood disorders, cohorts were also matched for specific mood disorder diagnoses. Cohorts were also matched for previous or concurrent use of specific medications with known association with COVID-19 including any antidepressant, fluvoxamine specifically,[12] any antipsychotic, and clozapine specifically.[13] Given the theoretical possibility that ACE-inhibitors and angiotensin receptor blockers might affect the pathogenesis of COVID-19 sequelae (despite the absence of association with incidence of and mortality from COVID-19),[14] these two classes of drugs were also included as covariates.

In total, 74 variables were used as covariates. More details including ICD-10 codes are provided in the Supplement. Cohorts were matched for all these variables, as described below.

### Outcomes

The outcome period was six months after COVID-19 diagnosis. The primary outcome was a composite of ICD-10 codes capturing the range of diagnostic codes that patients presenting with 'brain fog' might receive, as defined in our previous study.[1] Specifically the following codes were used: F01 ('Vascular dementia'), F02 ('Dementia in other disease classified elsewhere'), F03 ('Unspecified dementia'), F05 ('Delirium due to known physiological condition'), F06.8 ('Other specified mental disorders due to known physiological condition'), G30 ('Alzheimer's disease'), G31.0 ('Frontotemporal dementia'), G31.83 ('Dementia with Lewy bodies'), G31.84 ('Mild cognitive impairment'), G93.40 ('Encephalopathy, unspecified'), R40 ('Somnolence, stupor and coma'), R41 ('Other symptoms and signs involving cognitive functions and awareness'), or R48 ('Dyslexia and other symbolic dysfunction').

As secondary outcomes, the above ICD-10 codes were classified into 5 categories: dementia (F01-F03, G30, G31.0, or G31.83), delirium (F05), mild cognitive impairment (G31.84), encephalopathy (G93.40), and other cognitive deficits (any of the other codes: F06.8, R40, R41, or R48).

### Statistical analyses

Propensity score matching was used to create cohorts with matched baseline characteristics,[15] and carried out within the TriNetX network. Propensity score 1:1 matching used a greedy nearest neighbour matching approach with a caliper distance of 0.1 pooled standard deviations of the logit of the propensity score. Any characteristic with a standardized mean difference (SMD) between cohorts lower than 0.1 is considered well matched.[16] The incidence of each outcome was estimated using the Kaplan-Meier estimator. Comparisons between cohorts were made using a log-rank test. Hazard ratios (HR) with 95% confidence intervals were calculated using the Cox model. The proportional hazard assumption was tested using the generalized Schoenfeld approach. The E-value was used to quantify sensitivity of the findings to unmeasured confounders.[17] Statistical analyses were conducted in R version 3.6.3 except for the matching of cohorts which was performed within TriNetX. Statistical significance was set at two-sided p-values <0.05.

### Secondary analyses

To assess whether the association between phenytoin exposure and post-COVID cognitive impairment also affects the incidence towards the later phase of the follow-up, the statistical analysis was repeated with the weighted log-rank test with a Fleming-Harrington function with parameters p=0, q=1, which has optimal power to detect late effects of exposure on outcomes.[18]

Since levetiracetam and valproate are approved for the treatment of primary generalized epilepsy and focal epilepsies whereas phenytoin is mostly used for focal epilepsies, and since patients with intellectual disability are more likely to have focal than generalized epilepsy,[19] we ran a robustness analysis in which the epilepsy subtype (generalized epilepsy vs. other) and intellectual disabilities (F70-F79) were added to the list of covariates.

We tested the specificity of the association of phenytoin exposure with post-COVID cognitive deficit by including, as putative negative control outcomes, another eight common long-COVID clinical features as defined in our previous study,[1] namely chest/throat pain, headache, myalgia, other pain, abnormal breathing, abdominal symptoms, fatigue, and anxiety/depression. Each of them were captured with the same ICD-10 codes as used in our previous study (see Supplement for details).

As another secondary analysis, we compared patients with high phenytoin level (≥ 10 mcg/mL, i.e. above minimum therapeutic level) vs. low (< 10 mcg/mL, i.e. below therapeutic level). This exploratory analysis was unmatched as the sample size was too low to achieve appropriate matching and because both cohorts were receiving phenytoin thus reducing the potential for large confounding effects.

### Data availability

The TriNetX system returned the results of these analyses as csv files which were downloaded and archived. Data presented in this paper and the Supplement can be freely accessed at https://osf.io/wxtgu. Additionally, TriNetX will grant access to researchers if they have a specific concern (via the third-party agreement option).

## Results

A total of 668 individuals with SARS-CoV-2 infections had phenytoin recorded as a medication in their EHR within the 3 months preceding a diagnosis of COVID-19 or a confirmed PCR test for SARS-CoV-2. In the control cohorts, 4192 and 1344 individuals with SARS-CoV-2 infections had respectively levetiracetam and valproate recorded as a medication in their EHR within the 3 months preceding a diagnosis of COVID-19 or a confirmed PCR test for SARS-CoV-2. After 1:1 matching, 663 patients on phenytoin were compared to 663 patients on levetiracetam, and 505 patients on phenytoin were compared to 505 patients on valproate. Baseline characteristics of all cohorts are presented in Table 1 and Supplementary Tables 1-2. Adequate matching was achieved for all covariates in all comparisons.

Patients taking phenytoin at the time of COVID-19 were at a significantly lower risk of post-COVID cognitive deficits than a matched cohort of patients receiving levetiracetam (HR 0.78, 95% CI 0.63-0.97, p=0.024; E-value 1.66; no evidence of violation of proportionality assumption: p=0.42; Fig. 1A) or valproate (HR 0.73, 95% CI 0.58-0.93, p=0.011; E-value 1.79; no evidence of violation of proportionality assumption: p=0.77; Fig 1B) in the following six months. This relationship was sustained when primarily focusing on the later phase of follow-up (weighted log-rank p=0.021 when comparing to levetiracetam, and p=0.026 for valproate). It was also robust to adjustment for type of epilepsy and intellectual disabilities (HR 0.80, 95% CI 0.70-0.91, p=0.0005 for the comparison with levetiracetam and HR 0.86, 95% CI 0.75-0.99, p=0.039 for the comparison with valproate).

The association was found to be specific to post-COVID cognitive deficits as no significant associations were observed for any of the other long-COVID features (all p>0.05) except for a significantly lower risk of headache in those exposed to phenytoin compared to valproate (HR 0.59, 95% CI 0.38-0.93, p=0.02) but not compared to levetiracetam (see Supplementary Table 3).

The incidence of most components of the composite endpoint representing cognitive deficits were lower in the phenytoin cohort, with notable contributions from ‘other cognitive deficits’ (Fig. 2).

Patients with a therapeutic phenytoin level (n=240) were at a lower risk of post-COVID cognitive impairment than those with a subtherapeutic phenytoin level (n=219; the total number is less than the number of individuals on phenytoin because several patients on phenytoin did not have a level recorded during the pandemic), but this did not reach statistical significance (HR 0.76, 95% CI 0.55-1.05, p=0.091), except when focusing on the later phase of follow up (weighted log-rank p=0.021).

## Discussion

The findings in this study based on electronic health records show that receiving phenytoin (vs. levetiracetam or valproate) around the time of infection with SARS-CoV-2 is associated with a 22-27% lower risk of post-COVID cognitive deficits over the following six months compared to other antiepileptic drugs.

These findings provide the first empirical support for the hypothesis that exposure to a pharmacological inhibitor of RIPK signaling around the time of COVID-19 infection reduces the risk of post-COVID cognitive deficits. This in turn suggests that the pathogenesis of post-COVID cognitive deficits involves (at least in some cases) cerebral endotheliopathy. The downstream mechanism from endotheliopathy to cognitive impairment might follow the general pathophysiological pathway of small vessel diseases which is a leading cause of cognitive decline.[20] Experimental research on the role of RIPK signaling in COVID-related neuropathology has been limited to small vessels.[8] It is unclear if the same pathological process is responsible for large vessel ischaemic stroke after COVID-19 and the rate of ischaemic strokes was too low in our population for meaningful comparisons between matched cohorts (for completeness, the results in terms of ischaemic strokes are presented in Supplementary Fig. 1 showing non-statistically significant decreased rates in individuals on phenytoin compared to those on levetiracetam or valproate).

'Other cognitive symptoms' was the most common category of cognitive sequelae of COVID-19, which likely reflects the "brain fog", word finding difficulties or poor concentration reported in surveys of long-COVID.[1] The fact that (i) this is also the outcome with the largest difference in incidence (in absolute terms) in those receiving phenytoin, (ii) that the risk of delirium (which is typically restricted to the acute phase) appears unaffected by phenytoin, and (iii) that differences between cohorts is as notable or even more notable in later stages of the follow-up, suggest that endotheliopathy via RIPK signaling pathway might play a particularly important role in post-acute cognitive symptoms as part of long-COVID.

Our study is observational so no causal effect can be demonstrated and residual confounding (despite extensive matching) may persist. However, any unmeasured confounders would need to be associated with both the difference in exposure and post-COVID cognitive deficits with a relative risk of 1.66-fold each (i.e. the E-value) to explain away the observed association, which seems unlikely. In addition, it is worth noting that in the absence of COVID-19, levetiracetam is known to better preserve or even improve cognitive function[21,22] whereas phenytoin has recognised cognitive side effects.[23] The choice of control cohort might therefore have led to a conservative estimate of the association between phenytoin and post-COVID cognitive deficits, potentially offsetting any positive bias from unmeasured confounders.

There are other limitations to this study besides those general to studies based on EHR data (summarised in the Supplement). First, in the absence of a diagnostic code specific to the 'brain fog' reported by patients following COVID-19 infection, we used a pragmatic list of ICD-10 codes that are likely to capture this phenomenon and which were already used in a previous study.[1] Second, while we have hypothesised a specific mechanism, no direct information can be gleaned from EHR data to demonstrate the mechanism at play; prospective studies with measurements of biomarkers for endotheliopathy are needed to address this issue. In particular, further studies should assess whether phenytoin inhibits RIPK signaling at antiepileptic concentrations since in-vivo studies were conducted by injecting phenytoin intraperitoneally in mice.[10,11] Third, it is possible that the results do not apply to COVID-19 patients who do not have epilepsy. While this possibility cannot be ruled out from our data, endotheliopathy has been recognised as a core pathophysiological feature of COVID-19.[24]

These findings give support for a potential pathophysiological mechanism of post-COVID cognitive impairment, which is amenable to pharmacological intervention. The side effect profile of phenytoin makes it relatively unattractive for repurposing as a preventative treatment for post-COVID cognitive deficits. However, more selective inhibitors of the RIPK signaling pathway could merit testing to prevent the cognitive sequelae of COVID-19, or trialled as secondary prevention for patients with positive biomarkers for endotheliopathy.[25]

### Acknowledgements

The authors thank Arjune Sen and Masud Husain for helpful discussion.

### Funding

Work supported by the National Institute for Health Research (NIHR) Oxford Health Biomedical Research Centre (grant BRC-1215-20005), the Wolfson Foundation, and MQ Mental Health Research. MT is an NIHR Academic Clinical Fellow and NIHR Oxford Health BRC Senior Research Fellow. The views expressed are those of the authors and not necessarily those of the UK National Health Service, NIHR, or the UK Department of Health.

## Competing interests

The authors report no competing interests.

## Figure legends

**Fig. 1 - Kaplan-Meier curves for the incidence of cognitive deficits within the first 6 months after a diagnosis of COVID-19.** Comparison is made between patients on phenytoin and those on (A) levetiracetam, and (B) valproate. Shaded areas represent 95% confidence intervals.

**Fig. 2 - Cumulative 6-month incidence of different components of the primary composite endpoint**. Comparison is made between phenytoin and (A) levetiracetam, and (B) valproate. Note that because an individual may have more than one outcome, the sum of the incidence might exceed the incidence of the composite endpoint. Horizontal bars represent 95% confidence intervals. MCI=mild cognitive impairment.

ACCECPTED MANUSCRIPT

**Table 1 Baseline characteristics.** Only a subset of the most common and representative baseline characteristics is presented. All other baseline characteristics can be found in Supplementary Table 1 and 2. SMD = Standardised mean difference.

| | Comparison with levetiracetam | | | Comparison with valproate | | |
|---|---|---|---|---|---|---|
| | Phenytoin | Levetiracetam | SMD | Phenytoin | Valproate | SMD |
| Number | 663 | 663 | - | 505 | 505 | - |
| DEMOGRAPHICS | | | | | | |
| Age; mean (SD); y | 60.9 (15.7) | 61.3 (19.3) | 0.02 | 58.7 (15.6) | 57.9 (19.7) | 0.04 |
| Sex; n (%) | | | | | | |
| Female | 277 (41.8) | 268 (40.4) | 0.03 | 213 (42.2) | 209 (41.4) | 0.02 |
| Male | 386 (58.2) | 395 (59.6) | 0.03 | 292 (57.8) | 296 (58.6) | 0.02 |
| Race; n (%) | | | | | | |
| White | 369 (55.7) | 376 (56.7) | 0.02 | 300 (59.4) | 291 (57.6) | 0.04 |
| Black or African American | 212 (32.0) | 216 (32.6) | 0.01 | 145 (28.7) | 152 (30.1) | 0.03 |
| Unknown | 65 (9.8) | 57 (8.6) | 0.04 | 54 (10.7) | 51 (10.1) | 0.02 |
| COMORBIDITIES; n (%) | | | | | | |
| Overweight and obesity | 212 (32.0) | 212 (32.0) | 0 | 163 (32.3) | 159 (31.5) | 0.02 |
| Hypertensive disease | 463 (69.8) | 482 (72.7) | 0.06 | 340 (67.3) | 342 (67.7) | 0.008 |
| Type 2 diabetes mellitus | 213 (32.1) | 220 (33.2) | 0.02 | 172 (34.1) | 171 (33.9) | 0.004 |
| Asthma | 89 (13.4) | 86 (13.0) | 0.01 | 72 (14.3) | 73 (14.5) | 0.006 |
| Other chronic obstructive pulmonary disease | 116 (17.5) | 117 (17.6) | 0.004 | 89 (17.6) | 81 (16.0) | 0.04 |
| Nicotine dependence | 141 (21.3) | 149 (22.5) | 0.03 | 100 (19.8) | 92 (18.2) | 0.04 |
| Psychiatric comorbidities | | | | | | |
| Substance misuse | 197 (29.7) | 205 (30.9) | 0.03 | 142 (28.1) | 135 (26.7) | 0.03 |
| Psychotic disorders | 82 (12.4) | 86 (13.0) | 0.02 | 76 (15.1) | 70 (13.9) | 0.03 |
| Mood disorders | 247 (37.3) | 244 (36.8) | 0.009 | 207 (41.0) | 211 (41.8) | 0.02 |
| Anxiety disorders | 237 (35.7) | 246 (37.1) | 0.03 | 191 (37.8) | 185 (36.6) | 0.02 |
| Ischemic heart diseases | 210 (31.7) | 217 (32.7) | 0.02 | 152 (30.1) | 147 (29.1) | 0.02 |
| Other forms of heart disease | 317 (47.8) | 314 (47.4) | 0.009 | 241 (47.7) | 236 (46.7) | 0.02 |
| Chronic kidney disease (CKD) | 116 (17.5) | 114 (17.2) | 0.008 | 90 (17.8) | 90 (17.8) | 0 |
| Cerebral infarction | 121 (18.2) | 135 (20.4) | 0.05 | 91 (18.0) | 100 (19.8) | 0.05 |
| Unspecified dementia | 107 (16.1) | 105 (15.8) | 0.008 | 83 (16.4) | 83 (16.4) | 0 |
| MEDICATIONS; n (%) | | | | | | |
| Antidepressants | 311 (46.9) | 323 (48.7) | 0.04 | 247 (48.9) | 246 (48.7) | 0.004 |
| Antipsychotics | 208 (31.4) | 209 (31.5) | 0.003 | 182 (36.0) | 184 (36.4) | 0.008 |
| ACE inhibitors | 209 (31.5) | 227 (34.2) | 0.06 | 165 (32.7) | 151 (29.9) | 0.06 |
| Angiotensin II inhibitors | 97 (14.6) | 100 (15.1) | 0.01 | 67 (13.3) | 65 (12.9) | 0.01 |

ACCECPTED MANUSCRIPT

Graphical abstract

493x493mm (72 x 72 DPI)

## Supplementary Material

fcac206_Supplementary_Data

