# Supplementary material for: Exposure to phenytoin associates with a lower risk of post-COVID cognitive deficits: a cohort study
Source: Brain Commun. 2022 Aug 16;4(4):fcac206. doi: 10.1093/braincomms/fcac206 (PMC9384796; doi:10.1093/braincomms/fcac206)
Supplement: fcac206_Supplementary_Data [file fcac206_supplementary_data.pdf]

Supplementary Methods

TriNetX network

This section reproduces our previous description of the network.[1]

Legal and ethical status
TriNetX's Analytics network is compliant with the Health Insurance Portability and Accountability Act (HIPAA), the US federal law which protects the privacy and security of healthcare data. TriNetX is certified to the ISO 27001:2013 standard and maintains an Information Security Management System (ISMS) to ensure the protection of the healthcare data it has access to and to meet the requirements of the HIPAA Security Rule. Any data displayed on the TriNetX Platform in aggregate form, or any patient level data provided in a data set generated by the TriNetX Platform, only contains de-identified data as per the de-identification standard defined in Section §164.514(a) of the HIPAA Privacy Rule. The process by which the data is de-identified is attested to through a formal determination by a qualified expert as defined in Section §164.514(b)(1) of the HIPAA Privacy Rule. This formal determination by a qualified expert, refreshed in December 2020, supersedes the need for TriNetX's previous waiver from the Western Institutional Review Board (IRB). The network contains data that are provided by participating Health Care Organizations (HCOs), each of which represents and warrants that it has all necessary rights, consents, approvals and authority to provide the data to TriNetX under a Business Associate Agreement (BAA), so long as their name remains anonymous as a data source and their data are utilized for research purposes. The data shared through the TriNetX Platform are attenuated to ensure that they do not include sufficient information to facilitate the determination of which HCO contributed which specific information about a patient.

Acquisition of data, quality control, and other procedures
The data are stored onboard a TriNetX appliance – a physical server residing at the institution's data centre or a virtual hosted appliance. The TriNetX platform is a fleet of these appliances connected into a federated network able to broadcast queries to each appliance. Results are subsequently collected and aggregated.

Once the data are sent to the network, they are mapped to a standard and controlled set of clinical terminologies and undergo a data quality assessment including 'data cleaning' that rejects records which do not meet the TriNetX quality standards. HIPAA compliance of the clinical patient data is achieved using de-identification. Different data modalities are available in the network. They include demographics (coded to HL7 version 3 administrative standards), diagnoses (represented by ICD-10-CM codes), procedures (coded in ICD-10-PCS or CPT), measurements (coded to LOINC), and clinical drugs (represented as VA class and/or RxNorm). While extensive information is provided about patients' diagnoses and procedures, other variables (such as socioeconomic and lifetime factors are not comprehensively represented).

The data from a typical HCO generally go back around 7 years, with some going back 13 years. The data are continuously updated. HCOs update their data at various times, with most refreshing every 1, 2, or 4 weeks.

The data come primarily (>93%) from HCOs in the USA, with the remainder coming from India, Australia, Malaysia, Taiwan, Spain, UK, and Bulgaria. As noted above, to comply with legal frameworks and ethical guidelines guarding against data re-identification, the identity of participating HCOs and their individual contribution to each dataset are not disclosed to researchers.

Data quality assessment followed a standardised strategy wherein the data are reviewed for conformance (adherence to specified standards and formats), completeness (quantifying data presence or absence) and plausibility (believability of the data from a clinical perspective). There are pre-defined metrics for each of the above assessment categories. Results for these metrics are visualised and reviewed for each new site that joins the network as well as on an ongoing basis. Any identified issue is communicated to the data provider and resolved before continuing data collection.

The basic formatting of contributed data is also checked (e.g. to ensure that dates are properly represented). Records are checked against a list of required fields (e.g., patient identifier) and rejects those records for which the required information is missing. Referential integrity checking is done to ensure that data spanning multiple database tables can be successfully joined together. As the data are refreshed, changes in volume of data over

time is monitored to ensure data validity. At least one non-demographic fact for each patient is required for them to be counted in the dataset. Patient records with only demographics information are discarded.

The software also undergoes quality control. The engineers testing the software are independent from the engineers developing it. Each test code is checked by two independent testing engineers. Each piece of software is tested extensively against a range of synthetic data (i.e. generated for the purpose of testing) for which the expected output is established independently. If the software fails to return this output, then the software is deemed to have failed the test and is examined and modified accordingly. For statistical software (including that used for propensity score matching, for Kaplan-Meier analysis, etc), an additional quality control step is implemented. Two independent codes are written in two different programming languages (typically R and python) and the statistical results are compared. If discrepancies are identified, then the codes are deemed to have failed the test and are examined and modified accordingly. All the code is reviewed independently by another engineer.

The test strategy follows three levels of granularity:

1. Unit tests: These test specific blocks, or units, of code that perform specific actions (e.g. querying the database).
2. Integration tests: These ensure that different components are working together correctly.
3. End-to-end tests: These tests run the entire system and check the final output.

Some comments on advantages and disadvantages of EHR data
One advantage of EHR data, like those in TriNetX, over insurance claim data is that both insured and uninsured patients are included. An advantage of EHR data over survey data is that they represent the diagnostic rates in the population presenting to healthcare facilities. This provides an accurate account of the burden of specific diagnoses on healthcare systems. However, there are also limitations inherent to research using electronic health records,[2–4] including TriNetX:

1. Patients with acute or post-acute sequelae of COVID-19 but were not diagnosed are not included leading to underestimation of actual incidences.
2. Despite the matching and use of various comparison cohorts, there may well be residual confounding, particularly related to social and economic factors which are not well captured in EHR networks and which might influence outcomes post COVID-19.
3. We do not know which diagnoses were made in primary or secondary care or specialist facilities, nor by whom.
4. A patient may be seen in different HCOs for different parts of their care, and if one HCO is not part of the federated network then part of their medical records may not be available. Using a network of HCOs (rather than a single HCO) limits this possibility but does not fully remove it.
5. Since the data are presented as they are recorded, we cannot be sure that there has not been mis-recording of information, adding a degree of noise to the data.
6. Historical data before the start of EHRs (or the addition of an HCO to the network) may well be incomplete.

Definition of cohorts

The following medication codes were used in the definition of the primary and control cohorts:

- Phenytoin: RxNorm code 114477
- Levetiracetam: RxNorm code 8183
- Valproate: RxNorm code 40254

Confirmed COVID-19 diagnosis was based on the ICD-10 code U07.1. Positive PCR tests for COVID-19 was based on the presence in the individual's EHR of any of the following:

- Positive SARS-CoV-2 RNA in Respiratory specimen
- Positive SARS-CoV-2 RNA in Unspecified specimen
- Positive SARS-CoV-2 N gene in Respiratory specimen
- Positive SARS-CoV-2 N gene in Unspecified specimen
- Positive SARS-CoV-2 RdRp gene in Respiratory specimen
- Positive SARS-CoV-2 E gene in Respiratory specimen

- Positive SARS-CoV-2 E gene in Unspecified specimen
- Positive SARS-CoV-2 RNA panel in Respiratory specimen
- Positive SARS-CoV-2 RNA panel in Unspecified specimen
- Positive SARS-CoV-2 RNA in Nasopharynx
- Positive SARS coronavirus 2 and related RNA
- Positive SARS-related coronavirus RNA in Respiratory specimen
- Positive SARS coronavirus 2 ORF1ab in Respiratory specimen

Definition of covariates

To reduce the effect of confounding on associations, cohorts were matched for established or suspected risk factors for COVID-19[5–8] and for established risk factors for COVID-19 death[9] (taken to be risk factors of a more severe COVID-19 illness). These were the covariates used in our previous studies.[1,10,11] In this study, in addition to these covariates, cohorts were also matched for specific mood disorders (given the comorbidities with epilepsy), and for possible concurrent medications which are known to be associated with different incidence or outcomes of COVID-19, namely any antidepressant, fluvoxamine in particular, any antipsychotics, and clozapine in particular. As mentioned in the manuscript, given the theoretical possibility that ACE-inhibitors and angiotensin receptor blockers might affect the pathogenesis of COVID-19 sequelae (despite the absence of association with incidence of and mortality from COVID-19[12]), these two classes of drugs were also included as covariates. The following confounding factors were therefore included (with ICD-10/VA Class/RxNorm codes in brackets):

1) **Age** at the time of diagnosis.
2) **Sex** coded as female, male, or other.
3) **Race** encoded as 6 separate dichotomous variables: White (2106-3), Black or African American (2054-5), American Indian or Alaska Native (1002-5), Asian (2028-9), Native Hawaiian or Other Pacific Islander (2076-8), or Unknown Race (2131-1).
4) **Ethnicity** encoded as Hispanic or Latino (2135-2), Not Hispanic or Latino (2186-5), or Unknown Ethnicity.
5) **Socioeconomic deprivation** encoded as the ICD-10 code for Problems related to housing and economic circumstances (Z59).
6) **Obesity** encoded as one dichotomous variable and one categorical variable: Overweight and obesity (E66) and body mass index (categorised into < 25 kg/m^2, 25-30 kg/m^2, ≥ 30 kg/m^2 which are the WHO thresholds for not obese, pre-obese, and obesity).
7) **Hypertension** encoded as 2 dichotomous and 2 categorical variables: Hypertensive diseases (I10-I16), the now deprecated version that was used until 2018 Hypertension diseases (I10-I15), measurements of systolic blood pressure (categorised into < 140mmHg, 140-160mmHg, and ≥ 160mmHg), and diastolic blood pressure (categorised into < 90mmHg, 90-100mmHg, and ≥ 100mmHg). The blood pressure categories correspond to the absence of hypertension, stage 1 hypertension, and stage 2 (and over) hypertension as per the NICE guidelines.
8) **Diabetes mellitus** encoded as 2 dichotomous variables: Type 1 diabetes mellitus (E10) and Type 2 diabetes mellitus (E11).
9) **Chronic lower respiratory diseases** encoded by each sub-category of the corresponding ICD-10 group: Bronchitis, not specified as acute or chronic (J40), Simple and mucopurulent chronic bronchitis (J41), Unspecified chronic bronchitis (J42), Emphysema (J43), Other chronic obstructive pulmonary disease (J44), Asthma (J45), Bronchiectasis (J47).
10) **Nicotine dependence** encoded as the corresponding ICD-10 diagnosis (F17.2).
11) **Substance use disorders** encoded as the ICD-10 code for mental and behavioural disorders due to psychoactive substance use (F10-F19).
12) **Psychotic disorders** encoded as the ICD-10 code for schizophrenia, schizotypal, delusional, and other non-mood psychotic disorders (F20-F29).
13) **Mood disorders** encoded as a single variable (as well as individual codes, see below) with any of the ICD-10 code for mood disorders (F30-F39).
14) **Manic episode** (F30)
15) **Bipolar disorder** (F31)
16) **Major depressive disorder, single episode** (F32)
17) **Major depressive disorder, recurrent** (F33)
18) **Persistent mood disorders** (F34)
19) **Unspecified mood disorder** (F39)

20) **Anxiety disorders** encoded as the ICD-10 code for anxiety, dissociative, stress-related, somatoform and other nonpsychotic mental disorders (F40-F48)
21) **Heart diseases** encoded as 2 categorical variables: Ischaemic heart disease (I20-I25) and Other forms of heart disease (I30-I52).
22) **Chronic kidney disease** encoded as 2 dichotomous variables: Chronic kidney disease (N18) and Hypertensive chronic kidney disease (I12).
23) **Chronic liver disease** encoded as 8 categorical variables: Alcoholic liver disease (K70), Hepatic failure, not elsewhere classified (K72), Chronic hepatitis, not elsewhere classified (K73), Fibrosis and cirrhosis of liver (K74), Fatty (change of) liver, not elsewhere classified (K76.0), Chronic passive congestion of liver (K76.1), Portal hypertension (K76.6), Other specified diseases of liver (K76.8).
24) **Stroke** encoded as the dichotomous variable Cerebral infarction (I63) .
25) **Dementia** encoded as 6 dichotomous variables: Vascular dementia (F01), Dementia in other diseases classified elsewhere (F02), Unspecified dementia (F03), Alzheimer's disease (G30), Frontotemporal dementia (G31.0), and Dementia with Lewy bodies (G31.83).
26) **Cancer and haematological cancer in particular** encoded as 2 dichotomous variables: Neoplasms (C00-D49) and Malignant neoplasms of lymphoid, hematopoietic and related tissue (C81-C96).
27) **Organ transplant** encoded as 2 dichotomous variables: Renal Transplantation Procedures and Liver Transplantation Procedures.
28) **Rheumatoid arthritis** encoded as 2 dichotomous variables: Rheumatoid arthritis with rheumatoid factor (M05) and Other rheumatoid arthritis (M06).
29) **Lupus** encoded as a dichotomous variable corresponding ICD-10 code (M32).
30) **Psoriasis** encoded as a dichotomous variable corresponding ICD-10 code (L40).
31) **Disorders involving an immune mechanism** encoded as a dichotomous variable “Certain disorders involving the immune mechanism” (D80-D89).
32) **Antipsychotics** as a class, encoded as VA Class CN700
33) **Clozapine** encoded as RxNorm 2626
34) **Antidepressants** as a class, encoded as VA Class CN600
35) **Fluvoxamine** encoded as RxNorm 42355
36) **ACE inhibitors** as a class, encoded as VA Class CV800
37) **Angiotensin II inhibitors** as a class, encoded as VA Class CV805

Each individual code was considered a confounding factor in and of itself so that matching was achieved for each of them individually. For instance, matching was achieved for each subcategory (and not just for the whole category) of chronic lower respiratory diseases. For variables representing diagnoses and socioeconomic deprivation, an individual was considered positive if the diagnostic code was recorded at least once in their health record before the index event. For categorical variables representing measurements (i.e. BMI and blood pressures), all available measurements for all individuals were used and propensity score matching sought to define cohorts with similar numbers of measurements falling into each category.

Details on statistical analyses

Implementation details of propensity score matching
In propensity score matching, the propensity score was calculated using a logistic regression (implemented by the function LogisticRegression of the scikit-learn package in Python 3.7) including each of the covariates mentioned above. To eliminate the influence of ordering of records, the order of the records in the covariate matrix were randomised before matching.

Testing proportional hazards
The assumption that the hazards were proportional when accounting for the two phases was tested using the generalized Schoenfeld approach[13] implemented in the cox.zph function of the survival package (version 3.2.3) in R.

Weighted log-rank test
In the primary analysis, the standard log-rank test was used. As part of a secondary analysis aiming at investigating the risk of post-COVID cognitive deficits specifically in the post-acute phase, we used a weighted log-rank test with the Fleming-Harrington weight function with parameters p=0, and q=1 (i.e. the standard values of the parameters for the late-effect weighted logrank test[14]), as implemented in the comp function of the R survMisc package (version 0.5.5). This weighted log-rank test does not focus on a specific time window;

instead, it assigns increasing weight to events occurring increasingly late in the follow-up window (the weight at time *t* is proportional to the cumulative incidence up to time *t*).

Scenarios wherein both the standard log-rank test and weighted log-rank tests are significant (as is the case for the comparisons between phenytoin and levetiracetam or valproate) imply that the difference between the groups is not driven solely by differences occurring early in the follow-up.

Scenarios wherein the standard log-rank test results in a non-significant difference but the weighted log-rank test does (as is the case in the secondary analysis where high vs. low phenytoin levels are being compared) suggest that differences between the groups are mostly noticeable in the late phase of the follow-up.

Definition on negative controls

We tested the specificity of the association between phenytoin exposure and post-COVID cognitive deficits by assessing the association with other 'long-COVID' features. To do so, we followed the exact same analytic plan as for the primary analysis but changed the outcome to each of the other 8 long-COVID features that were used in our previous study based on the same dataset.[1]

Specifically, the following ICD-10 codes (with the ICD-10 labels in brackets) were used to define outcomes:

1) **Chest/Throat pain**: R07 ('Pain in throat and chest').
2) **Abnormal breathing**: R06 ('Abnormalities of breathing').
3) **Abdominal symptoms**: R10 ('Abdominal and pelvic pain'), R19.4 ('Change in bowel habit'), or R19.7 ('Diarrhoea, unspecified').
4) **Fatigue**: G93.3 ('Postviral fatigue syndrome') or R53 ('Malaise and fatigue').
5) **Anxiety/Depression**: F30-F39 ('Mood disorders') or F40-F48 ('Anxiety, dissociative, stress-related, somatoform and other nonpsychotic mental disorders'). F30-39 also encompasses diagnoses other than depression (e.g. mania), but these comprise only a small fraction of cases and so we use Anxiety/Depression for convenience.
6) **Pain**: G89 ('Pain not elsewhere specified') or R52 ('Pain, unspecified').
7) **Headache**: R51 ('Headache'), G43 ('Migraine'), or G44 ('Other headache syndrome').
8) **Myalgia**: M79.1 ('Myalgia') or M60 ('Myositis'). Inclusion of the latter category was intended to capture those patients who received a specific diagnosis when presenting with myalgia.

Supplementary Tables

Supplementary Table 1 – Baseline characteristics for the phenytoin and levetiracetam cohorts before and after matching.

	Before matching			After matching		
	**Phenytoin**	**Levetiracetam**	**SMD**	**Phenytoin**	**Levetiracetam**	**SMD**
Number	668	4192	-	663	663	-
DEMOGRAPHICS						
Age; mean (SD); y	60.9 (15.6)	49.7 (23.9)	0.6	60.9 (15.7)	61.3 (19.3)	0.02
Sex; n (%)						
Female	279 (41.8)	2112 (50.4)	0.2	277 (41.8)	268 (40.4)	0.03
Male	389 (58.2)	2080 (49.6)	0.2	386 (58.2)	395 (59.6)	0.03
Other	0 (0.0)	0 (0.0)	NA	0 (0.0)	0 (0.0)	NA
Race; n (%)						
White	372 (55.7)	2335 (55.7)	3.00E-04	369 (55.7)	376 (56.7)	0.02
Black or African American	213 (31.9)	1250 (29.8)	0.04	212 (32.0)	216 (32.6)	0.01
Asian	13 (1.9)	83 (2.0)	0.002	13 (2.0)	10 (1.5)	0.03
American Indian or Alaska Native	10 (1.5)	22 (0.5)	0.1	10 (1.5)	10 (1.5)	0
Native Hawaiian or Other Pacific Islander	10 (1.5)	10 (0.2)	0.1	10 (1.5)	10 (1.5)	0
Unknown	66 (9.9)	498 (11.9)	0.06	65 (9.8)	57 (8.6)	0.04
Ethnicity; n (%)						
Hispanic or Latino	60 (9.0)	497 (11.9)	0.09	60 (9.1)	55 (8.3)	0.03
Not Hispanic of Latino	421 (63.0)	2839 (67.7)	0.1	420 (63.3)	436 (65.8)	0.05
Unknown	187 (28.0)	856 (20.4)	0.2	183 (27.6)	172 (25.9)	0.04
Socioeconomic deprivation; n (%)	32 (4.8)	207 (4.9)	0.007	32 (4.8)	37 (5.6)	0.03
COMORBIDITIES; n (%)						
Overweight and obesity	216 (32.3)	1173 (28.0)	0.09	212 (32.0)	212 (32.0)	0
Hypertensive disease	468 (70.1)	2501 (59.7)	0.2	463 (69.8)	482 (72.7)	0.06
Diabetes mellitus						
Type 1 diabetes mellitus	34 (5.1)	257 (6.1)	0.05	34 (5.1)	34 (5.1)	0
Type 2 diabetes mellitus	214 (32.0)	1370 (32.7)	0.01	213 (32.1)	220 (33.2)	0.02
Chronic lower respiratory diseases						
Bronchitis; not specified as acute or chronic	52 (7.8)	314 (7.5)	0.01	51 (7.7)	51 (7.7)	0
Simple and mucopurulent chronic bronchitis	10 (1.5)	46 (1.1)	0.04	10 (1.5)	13 (2.0)	0.03
Unspecified chronic bronchitis	10 (1.5)	57 (1.4)	0.01	10 (1.5)	10 (1.5)	0
Emphysema	36 (5.4)	209 (5.0)	0.02	36 (5.4)	38 (5.7)	0.01
Other chronic obstructive pulmonary disease	118 (17.7)	633 (15.1)	0.07	116 (17.5)	117 (17.6)	0.004
Asthma	89 (13.3)	801 (19.1)	0.2	89 (13.4)	86 (13.0)	0.01
Bronchiectasis	10 (1.5)	82 (2.0)	0.04	10 (1.5)	10 (1.5)	0
Nicotine dependence	143 (21.4)	764 (18.2)	0.08	141 (21.3)	149 (22.5)	0.03
Psychiatric comorbidities						
Substance misuse	200 (29.9)	1147 (27.4)	0.06	197 (29.7)	205 (30.9)	0.03
Psychotic disorders	83 (12.4)	381 (9.1)	0.1	82 (12.4)	86 (13.0)	0.02

Mood disorders	250 (37.4)	1501 (35.8)	0.03	247 (37.3)	244 (36.8)	0.009
Manic episode	10 (1.5)	23 (0.5)	0.09	10 (1.5)	10 (1.5)	0
Bipolar disorder	42 (6.3)	264 (6.3)	4.00E-04	42 (6.3)	43 (6.5)	0.006
Major depressive disorder; single episode	214 (32.0)	1306 (31.2)	0.02	212 (32.0)	213 (32.1)	0.003
Major depressive disorder; recurrent	71 (10.6)	354 (8.4)	0.07	69 (10.4)	72 (10.9)	0.01
Persistent mood disorders	37 (5.5)	153 (3.6)	0.09	36 (5.4)	33 (5.0)	0.02
Unspecified mood disorder	34 (5.1)	249 (5.9)	0.04	34 (5.1)	32 (4.8)	0.01
Anxiety disorders	239 (35.8)	1574 (37.5)	0.04	237 (35.7)	246 (37.1)	0.03
Heart disease						
Ischemic heart diseases	210 (31.4)	1110 (26.5)	0.1	210 (31.7)	217 (32.7)	0.02
Other forms of heart disease	318 (47.6)	2068 (49.3)	0.03	317 (47.8)	314 (47.4)	0.009
Chronic kidney diseases						
Chronic kidney disease (CKD)	116 (17.4)	924 (22.0)	0.1	116 (17.5)	114 (17.2)	0.008
Hypertensive chronic kidney disease	78 (11.7)	634 (15.1)	0.1	78 (11.8)	80 (12.1)	0.009
Chronic liver disease						
Alcoholic liver disease	10 (1.5)	120 (2.9)	0.09	10 (1.5)	13 (2.0)	0.03
Hepatic failure; not elsewhere classified	13 (1.9)	154 (3.7)	0.1	13 (2.0)	10 (1.5)	0.03
Chronic hepatitis; not elsewhere classified	10 (1.5)	24 (0.6)	0.09	10 (1.5)	10 (1.5)	0
Fibrosis and cirrhosis of liver	11 (1.6)	185 (4.4)	0.2	11 (1.7)	14 (2.1)	0.03
Fatty (change of) liver; not elsewhere classified	32 (4.8)	282 (6.7)	0.08	32 (4.8)	33 (5.0)	0.007
Chronic passive congestion of liver	10 (1.5)	63 (1.5)	5.00E-04	10 (1.5)	10 (1.5)	0
Portal hypertension	10 (1.5)	89 (2.1)	0.05	10 (1.5)	10 (1.5)	0
Other specified diseases of liver	25 (3.7)	230 (5.5)	0.08	25 (3.8)	22 (3.3)	0.02
Cerebral infarction	121 (18.1)	897 (21.4)	0.08	121 (18.2)	135 (20.4)	0.05
Dementia						
Vascular dementia	31 (4.6)	183 (4.4)	0.01	31 (4.7)	33 (5.0)	0.01
Dementia in other diseases classified elsewhere	39 (5.8)	209 (5.0)	0.04	39 (5.9)	43 (6.5)	0.03
Unspecified dementia	108 (16.2)	487 (11.6)	0.1	107 (16.1)	105 (15.8)	0.008
Alzheimer disease	22 (3.3)	134 (3.2)	0.005	22 (3.3)	20 (3.0)	0.02
Frontotemporal dementia	10 (1.5)	10 (0.2)	0.1	10 (1.5)	0 (0.0)	0.2
Dementia with Lewy bodies	10 (1.5)	11 (0.3)	0.1	10 (1.5)	10 (1.5)	0
Neoplasms						
Neoplasms (any)	200 (29.9)	1341 (32.0)	0.04	198 (29.9)	203 (30.6)	0.02
Haematological cancer	18 (2.7)	115 (2.7)	0.003	18 (2.7)	12 (1.8)	0.06
Organ transplant						
Renal Transplantation Procedures	0 (0.0)	18 (0.4)	0.09	0 (0.0)	0 (0.0)	NA
Liver Transplantation Procedures	0 (0.0)	15 (0.4)	0.08	0 (0.0)	0 (0.0)	NA
Psoriasis	10 (1.5)	72 (1.7)	0.02	10 (1.5)	10 (1.5)	0
Rheumatoid arthritis						
Rheumatoid arthritis with rheumatoid factor	10 (1.5)	21 (0.5)	0.1	10 (1.5)	0 (0.0)	0.2
Other rheumatoid arthritis	11 (1.6)	127 (3.0)	0.09	11 (1.7)	10 (1.5)	0.01
Systemic lupus erythematosus (SLE)	12 (1.8)	85 (2.0)	0.02	11 (1.7)	10 (1.5)	0.01
Disorders involving the immune mechanism	24 (3.6)	288 (6.9)	0.1	23 (3.5)	29 (4.4)	0.05

MEDICATIONS; n (%)						
Antidepressants	313 (46.9)	1967 (46.9)	0.001	311 (46.9)	323 (48.7)	0.04
Fluvoxamine	10 (1.5)	20 (0.5)	0.1	10 (1.5)	10 (1.5)	0
Antipsychotics	209 (31.3)	1276 (30.4)	0.02	208 (31.4)	209 (31.5)	0.003
Clozapine	10 (1.5)	14 (0.3)	0.1	10 (1.5)	10 (1.5)	0
ACE inhibitors	213 (31.9)	1163 (27.7)	0.09	209 (31.5)	227 (34.2)	0.06
Angiotensin II inhibitors	97 (14.5)	606 (14.5)	0.002	97 (14.6)	100 (15.1)	0.01

Supplementary Table 2 – Baseline characteristics for the phenytoin and valproate cohorts before and after matching.

	Before matching			After matching		
	**Phenytoin**	**Valproate**	**SMD**	**Phenytoin**	**Valproate**	**SMD**
Number	668	1344	-	505	505	-
DEMOGRAPHICS						
Age; mean (SD); y	60.9 (15.6)	48.0 (22.2)	0.7	58.7 (15.6)	57.9 (19.7)	0.04
Sex; n (%)						
Female	279 (41.8)	577 (42.9)	0.02	213 (42.2)	209 (41.4)	0.02
Male	389 (58.2)	767 (57.1)	0.02	292 (57.8)	296 (58.6)	0.02
Other	0 (0.0)	0 (0.0)	NA	0 (0.0)	0 (0.0)	NA
Race; n (%)						
White	372 (55.7)	864 (64.3)	0.2	300 (59.4)	291 (57.6)	0.04
Black or African American	213 (31.9)	309 (23.0)	0.2	145 (28.7)	152 (30.1)	0.03
Asian	13 (1.9)	21 (1.6)	0.03	10 (2.0)	10 (2.0)	0
American Indian or Alaska Native	10 (1.5)	10 (0.7)	0.07	0 (0.0)	10 (2.0)	0.2
Native Hawaiian or Other Pacific Islander	10 (1.5)	0 (0.0)	0.2	0 (0.0)	0 (0.0)	NA
Unknown	66 (9.9)	144 (10.7)	0.03	54 (10.7)	51 (10.1)	0.02
Ethnicity; n (%)						
Hispanic or Latino	60 (9.0)	128 (9.5)	0.02	38 (7.5)	37 (7.3)	0.008
Not Hispanic of Latino	421 (63.0)	920 (68.5)	0.1	331 (65.5)	339 (67.1)	0.03
Unknown	187 (28.0)	296 (22.0)	0.1	136 (26.9)	129 (25.5)	0.03
Socioeconomic deprivation; n (%)	32 (4.8)	93 (6.9)	0.09	26 (5.1)	26 (5.1)	0
COMORBIDITIES; n (%)						
Overweight and obesity	216 (32.3)	410 (30.5)	0.04	163 (32.3)	159 (31.5)	0.02
Hypertensive disease	468 (70.1)	729 (54.2)	0.3	340 (67.3)	342 (67.7)	0.008
Diabetes mellitus						
Type 1 diabetes mellitus	34 (5.1)	84 (6.2)	0.05	26 (5.1)	24 (4.8)	0.02
Type 2 diabetes mellitus	214 (32.0)	430 (32.0)	9.00E-04	172 (34.1)	171 (33.9)	0.004
Chronic lower respiratory diseases						
Bronchitis; not specified as acute or chronic	52 (7.8)	101 (7.5)	0.01	40 (7.9)	38 (7.5)	0.01
Simple and mucopurulent chronic bronchitis	10 (1.5)	10 (0.7)	0.07	10 (2.0)	10 (2.0)	0
Unspecified chronic bronchitis	10 (1.5)	19 (1.4)	0.007	10 (2.0)	10 (2.0)	0
Emphysema	36 (5.4)	52 (3.9)	0.07	24 (4.8)	21 (4.2)	0.03
Other chronic obstructive pulmonary disease	118 (17.7)	210 (15.6)	0.05	89 (17.6)	81 (16.0)	0.04
Asthma	89 (13.3)	263 (19.6)	0.2	72 (14.3)	73 (14.5)	0.006
Bronchiectasis	10 (1.5)	14 (1.0)	0.04	10 (2.0)	10 (2.0)	0
Nicotine dependence	143 (21.4)	238 (17.7)	0.09	100 (19.8)	92 (18.2)	0.04
Psychiatric comorbidities						
Substance misuse	200 (29.9)	352 (26.2)	0.08	142 (28.1)	135 (26.7)	0.03
Psychotic disorders	83 (12.4)	283 (21.1)	0.2	76 (15.1)	70 (13.9)	0.03
Mood disorders	250 (37.4)	658 (49.0)	0.2	207 (41.0)	211 (41.8)	0.02
Manic episode	10 (1.5)	26 (1.9)	0.03	10 (2.0)	10 (2.0)	0

Bipolar disorder	42 (6.3)	273 (20.3)	0.4	42 (8.3)	38 (7.5)	0.03
Major depressive disorder; single episode	214 (32.0)	487 (36.2)	0.09	174 (34.5)	174 (34.5)	0
Major depressive disorder; recurrent	71 (10.6)	173 (12.9)	0.07	59 (11.7)	58 (11.5)	0.006
Persistent mood disorders	37 (5.5)	70 (5.2)	0.01	24 (4.8)	25 (5.0)	0.009
Unspecified mood disorder	34 (5.1)	195 (14.5)	0.3	32 (6.3)	34 (6.7)	0.02
Anxiety disorders	239 (35.8)	615 (45.8)	0.2	191 (37.8)	185 (36.6)	0.02
Heart disease						
Ischemic heart diseases	210 (31.4)	303 (22.5)	0.2	152 (30.1)	147 (29.1)	0.02
Other forms of heart disease	318 (47.6)	603 (44.9)	0.05	241 (47.7)	236 (46.7)	0.02
Chronic kidney diseases						
Chronic kidney disease (CKD)	116 (17.4)	233 (17.3)	8.00E-04	90 (17.8)	90 (17.8)	0
Hypertensive chronic kidney disease	78 (11.7)	165 (12.3)	0.02	61 (12.1)	58 (11.5)	0.02
Chronic liver disease						
Alcoholic liver disease	10 (1.5)	18 (1.3)	0.01	10 (2.0)	10 (2.0)	0
Hepatic failure; not elsewhere classified	13 (1.9)	25 (1.9)	0.006	10 (2.0)	11 (2.2)	0.01
Chronic hepatitis; not elsewhere classified	10 (1.5)	10 (0.7)	0.07	10 (2.0)	10 (2.0)	0
Fibrosis and cirrhosis of liver	11 (1.6)	21 (1.6)	0.007	10 (2.0)	10 (2.0)	0
Fatty (change of) liver; not elsewhere classified	32 (4.8)	77 (5.7)	0.04	26 (5.1)	24 (4.8)	0.02
Chronic passive congestion of liver	10 (1.5)	11 (0.8)	0.06	10 (2.0)	10 (2.0)	0
Portal hypertension	10 (1.5)	10 (0.7)	0.07	10 (2.0)	10 (2.0)	0
Other specified diseases of liver	25 (3.7)	53 (3.9)	0.01	17 (3.4)	12 (2.4)	0.06
Cerebral infarction	121 (18.1)	205 (15.3)	0.08	91 (18.0)	100 (19.8)	0.05
Dementia						
Vascular dementia	31 (4.6)	73 (5.4)	0.04	26 (5.1)	27 (5.3)	0.009
Dementia in other diseases classified elsewhere	39 (5.8)	96 (7.1)	0.05	36 (7.1)	40 (7.9)	0.03
Unspecified dementia	108 (16.2)	183 (13.6)	0.07	83 (16.4)	83 (16.4)	0
Alzheimer disease	22 (3.3)	54 (4.0)	0.04	22 (4.4)	20 (4.0)	0.02
Frontotemporal dementia	10 (1.5)	10 (0.7)	0.07	10 (2.0)	10 (2.0)	0
Dementia with Lewy bodies	10 (1.5)	10 (0.7)	0.07	10 (2.0)	10 (2.0)	0
Neoplasms						
Neoplasms (any)	200 (29.9)	384 (28.6)	0.03	148 (29.3)	151 (29.9)	0.01
Haematological cancer	18 (2.7)	18 (1.3)	0.1	10 (2.0)	10 (2.0)	0
Organ transplant						
Renal Transplantation Procedures	0 (0.0)	10 (0.7)	0.1	0 (0.0)	0 (0.0)	NA
Liver Transplantation Procedures	0 (0.0)	0 (0.0)	NA	0 (0.0)	0 (0.0)	NA
Psoriasis	10 (1.5)	19 (1.4)	0.007	10 (2.0)	10 (2.0)	0
Rheumatoid arthritis						
Rheumatoid arthritis with rheumatoid factor	10 (1.5)	10 (0.7)	0.07	10 (2.0)	10 (2.0)	0
Other rheumatoid arthritis	11 (1.6)	35 (2.6)	0.07	10 (2.0)	10 (2.0)	0
Systemic lupus erythematosus (SLE)	12 (1.8)	21 (1.6)	0.02	10 (2.0)	10 (2.0)	0
Disorders involving the immune mechanism	24 (3.6)	55 (4.1)	0.03	17 (3.4)	20 (4.0)	0.03
MEDICATIONS; n (%)						
Antidepressants	313 (46.9)	746 (55.5)	0.2	247 (48.9)	246 (48.7)	0.004

Fluvoxamine	10 (1.5)	21 (1.6)	0.005	10 (2.0)	10 (2.0)	0
Antipsychotics	209 (31.3)	671 (49.9)	0.4	182 (36.0)	184 (36.4)	0.008
Clozapine	10 (1.5)	30 (2.2)	0.05	10 (2.0)	10 (2.0)	0
ACE inhibitors	213 (31.9)	364 (27.1)	0.1	165 (32.7)	151 (29.9)	0.06
Angiotensin II inhibitors	97 (14.5)	150 (11.2)	0.1	67 (13.3)	65 (12.9)	0.01

Supplementary Table 3 – Comparison between exposure to phenytoin and exposure to levetiracetam or valproate in terms of the 6-month risk of other long-COVID features used as negative controls.

	Comparison with levetiracetam		Comparison with valproate	
	**HR (95% CI)**	**p**	**HR (95% CI)**	**p**
**Anxiety/Depression**	0.92 (0.74-1.14)	0.43	0.89 (0.70-1.13)	0.34
**Chest/Throat pain**	1.22 (0.83-1.80)	0.32	0.92 (0.61-1.40)	0.70
**Abnormal breathing**	0.75 (0.56-1.00)	0.051	0.72 (0.51-1.00)	0.052
**Myalgia**	0.62 (0.27-1.46)	0.27	2.78 (0.71-10.86)	0.12
**Fatigue**	0.82 (0.63-1.06)	0.13	0.94 (0.69-1.27)	0.67
**Headache**	0.70 (0.46-1.06)	0.091	0.59 (0.38-0.93)	0.02
**Abdominal symptoms**	1.09 (0.80-1.48)	0.60	1.29 (0.90-1.85)	0.17
**Other pain**	0.84 (0.60-1.18)	0.32	0.88 (0.61-1.27)	0.50

Supplementary Figures

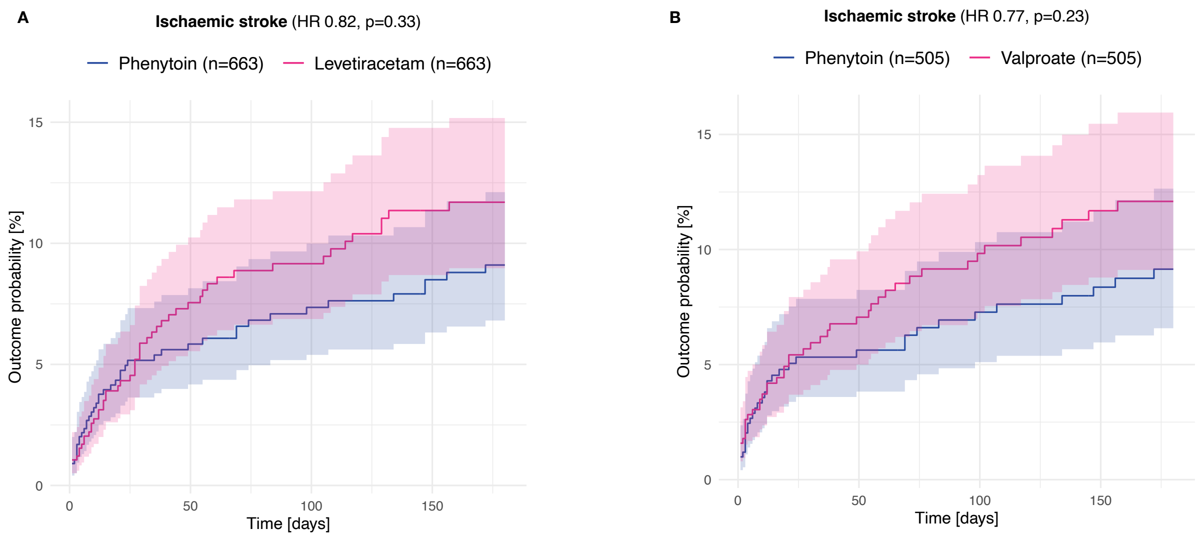

Supplementary Fig. 1 - Kaplan-Meier curves for the incidence of ischaemic strokes within the first 6 months after a diagnosis of COVID-19 comparing patients on phenytoin to those on (A) levetiracetam, and (B) valproate.

References

1 Taquet M, Dercon Q, Luciano S, Geddes JR, Husain M, Harrison PJ. Incidence, co-occurrence, and evolution of long-COVID features: A 6-month retrospective cohort study of 273,618 survivors of COVID-19. *PLoS Med* 2021; **18**: e1003773.

2 Casey JA, Schwartz BS, Stewart WF, Adler NE. Using Electronic Health Records for Population Health Research: A Review of Methods and Applications. *Annu Rev Public Health* 2016; **37**: 61–81.

3 Cowie MR, Blomster JI, Curtis LH, *et al.* Electronic health records to facilitate clinical research. *Clin Res Cardiol* 2017; **106**: 1–9.

4 Jetley G, Zhang H. Electronic health records in IS research: Quality issues, essential thresholds and remedial actions. *Decis Support Syst* 2019; **126**: 113137.

5 de Lusignan S, Dorward J, Correa A, *et al.* Risk factors for SARS-CoV-2 among patients in the Oxford Royal College of General Practitioners Research and Surveillance Centre primary care network: a cross-sectional study. *Lancet Infect Dis* 2020; published online May 15. DOI:10.1016/S1473-3099(20)30371-6.

6 Zhang J-J, Dong X, Cao Y-Y, *et al.* Clinical characteristics of 140 patients infected with SARS-CoV-2 in Wuhan, China. *Allergy* 2020. https://onlinelibrary.wiley.com/doi/abs/10.1111/all.14238.

7 Chen N, Zhou M, Dong X, *et al.* Epidemiological and clinical characteristics of 99 cases of 2019 novel coronavirus pneumonia in Wuhan, China: a descriptive study. *Lancet* 2020; **395**: 507–13.

8 Wang QQ, Kaelber DC, Xu R, Volkow ND. COVID-19 risk and outcomes in patients with substance use disorders: analyses from electronic health records in the United States. *Mol Psychiatry* 2021; **26**: 30–9.

9 Williamson EJ, Walker AJ, Bhaskaran K, *et al.* OpenSAFELY: factors associated with COVID-19 death in 17 million patients. *Nature* 2020; published online July 8. DOI:10.1038/s41586-020-2521-4.

10 Taquet M, Luciano S, Geddes JR, Harrison PJ. Bidirectional associations between COVID-19 and psychiatric disorder: retrospective cohort studies of 62 354 COVID-19 cases in the USA. *Lancet Psychiatry* 2021; **8**: 130–40.

11 Taquet M, Geddes JR, Husain M, Luciano S, Harrison PJ. 6-month neurological and psychiatric outcomes in 236 379 survivors of COVID-19: a retrospective cohort study using electronic health records. *Lancet Psychiatry* 2021; **8**: 416–27.

12 Fosbøl EL, Butt JH, Østergaard L, *et al.* Association of Angiotensin-Converting Enzyme Inhibitor or Angiotensin Receptor Blocker Use With COVID-19 Diagnosis and Mortality. *JAMA* 2020; **324**: 168–77.

13 Grambsch PM, Therneau TM. Proportional hazards tests and diagnostics based on weighted residuals. *Biometrika* 1994; **81**: 515.

14 Royston P, B Parmar MK. A simulation study comparing the power of nine tests of the treatment effect in randomized controlled trials with a time-to-event outcome. *Trials* 2020; **21**: 315.
